# Expression and Characterization of the *RKOD* DNA Polymerase in *Pichia pastoris*

**DOI:** 10.1371/journal.pone.0131757

**Published:** 2015-07-02

**Authors:** Fei Wang, Shuntang Li, Hui Zhao, Lu Bian, Liang Chen, Zhen Zhang, Xing Zhong, Lixin Ma, Xiaolan Yu

**Affiliations:** 1 Hubei Collaborative Innovation Center for Green Transformation of Bio-resources, Hubei Key Laboratory of Industrial Biotechnology, College of Life Sciences, Hubei University, Wuhan, People’s Republic of China; 2 Department of Bioengineering, Zhixing College of HuBei University, Wuhan, People’s Republic of China; Russian Academy of Sciences, Institute for Biological Instrumentation, RUSSIAN FEDERATION

## Abstract

The present study assessed high-level expression of the *KOD* DNA polymerase in *Pichia pastoris*. *Thermococcus kodakaraensis KOD1* is a DNA polymerase that is widely used in PCR. The DNA coding sequence of *KOD* was optimized based on the codon usage bias of *P*. *pastoris* and synthesized by overlapping PCR, and the nonspecific DNA-binding protein *Sso7d* from the crenarchaeon *Sulfolobus solfataricus* was fused to the C-terminus of *KOD*. The resulting novel gene was cloned into a pHBM905A vector and introduced into *P*. *pastoris* GS115 for secretory expression. The yield of the target protein reached approximately 250 mg/l after a 6-d induction with 1% (v/v) methanol in shake flasks. This yield is much higher than those of other DNA polymerases expressed heterologously in *Escherichia coli*. The recombinant enzyme was purified, and its enzymatic features were studied. Its specific activity was 19,384 U/mg. The recombinant *KOD* expressed in *P*. *pastoris* exhibited excellent thermostability, extension rate and fidelity. Thus, this report provides a simple, efficient and economic approach to realize the production of a high-performance thermostable DNA polymerase on a large scale. This is the first report of the expression in yeast of a DNA polymerase for use in PCR.

## Introduction

More than 50 DNA polymerases have been cloned and sequenced from various organisms. Most native thermostable enzymes are synthesized at low levels by thermophilic bacteria and consequently are difficult to purify. Several thermostable DNA polymerases have been produced in a biologically active form using *Escherichia coli* and baculovirus systems. The *Tay*, *Pfu*, *Pwo* and *Taq* DNA polymerases were expressed in *E*. *coli* at expression levels of approximately 2.25 mg/l, 24 mg/l, 26.6 mg/l, and 95 mg/l, respectively [1–3]. However, the lower soluble yields were not satisfactory. The *Pfu* DNA polymerase was also expressed as a secreted protein in *Spodoptera frugiperda* cells (sf-9) and *Trichoplusia ni* cells (hi-5) at expression levels of approximately 100 mg/l and 134 mg/l, respectively [4]. Baculovirus expression enables the production of commercial quantities but is more difficult and expensive than bacterial expression.

Increasing the output and simplifying the purification steps of thermostable DNA polymerases have recently been emphasized [5]. *Pichia pastoris* is an established protein expression host that is mainly applied for the production of biopharmaceuticals and industrial enzymes. This methylotrophic yeast can grow to very high cell densities and thus represents a unique production system for strong and tightly regulated promoters that can produce gram amounts of recombinant proteins per liter of culture both intracellularly and in a secretory fashion [6].

The KOD DNA polymerase from the *archaeon Pyrococcus sp*. *strain KOD1* exhibits 5-fold higher extension rate (100 to 130 nucleotides/s) and 10- to 15-fold higher processivity (persistence of sequential nucleotide polymerization) compared to the DNA polymerase from *Pyrococcus furiosus* (Pfu DNA polymerase), and the mutation frequency (3.5 ×10^−3^) was similar to Pfu DNA polymerase. Due to these characteristics, KOD DNA polymerase can be used to perform more accurate PCR with a shorter reaction time. In the present study, we report the high-level secretory expression of *KOD* DNA polymerase in *P*. *pastoris* for the first time.

The dsDNA binding protein Sso7d from *Sulfolobus solfataricus* has been reported to significantly enhance the processivity of *KOD* polymerase [7]. Therefore, in this study, the *Sso7d* gene was fused to the *KOD* C-terminus to enhance the processivity.

## Materials and Methods

### Strains, vectors, media and reagents

*P*. *pastoris* GS115 was obtained from Invitrogen (USA). *E*. *coli* XL10-Gold was purchased from Stratagene (USA). The pMD18-T vector was purchased from Takara (JPN). The yeast expression vector pHBM905A (*ColE1 ori*, *Amp* ^*r*^, *Kan* ^*r*^, *HIS4*, *P*_*AOX1*_, *T*_*AOX1*_) was synthesized previously in our laboratory [8]. The vector pBAD-His-GFP was constructed previously [9]. Luria-Bertani (LB) medium was prepared for the cultivation of *E*. *coli* as described in the Manual of Molecular Cloning [10]. For yeast culture, buffered glycerol-complex medium (BMGY), buffered methanol-complex medium (BMMY) and minimal dextrose medium (MD) were prepared as specified in the *Pichia* Expression Kit (Invitrogen, USA). Endoglycosidase H (Endo H) was purchased from NEB (USA). The DNA Gel Extraction Kit was from V-gene (USA). Restriction endonucleases (*Cop*I, *Not*I, and *Sal*I), *Dpn*I, T4 DNA polymerase, and the *Prime Star* DNA polymerase were purchased from TaKaRa (JPN). *KOD* plus was purchased from Toyobo (JPN). dATP, dCTP, dGTP, and dTTP were from Thermo Scientific (USA). [α-^32^P]-dCTP was from PerkinElmer (USA). Primers were synthesized by Sangon Biotech (China). All other reagents used in this study were of analytical grade.

### Design and synthesis of the KOD and Sso7d genes

The DNA coding sequences of *KOD* from *Pyrococcus sp*. *KOD1* (Gene Bank: D29671) and Sso7d from *S*. *solfataricus P2* (Gene ID: 1454006) were optimized to match the codon usage preference of *Pichia pastoris*. *Sso7d* was fused to the C-terminus of *KOD*. To synthesize this modified ORF, 72 oligonucleotides (S1 Table) were designed for overlapping PCR using the DNAWorks program (http://helixweb.nih.gov/dnaworks). The gene synthesis procedure was performed as described previously [11]. The novel DNA fragment was cloned into pMD18-T and confirmed by DNA sequencing (Sangon, China).

### Constructing the expression vector for *KOD* expression in *Pichia pastoris* GS115 and *E*. *coli*

For expression in *Pichia pastoris*, the target gene was amplified from the recombinant T-vector using the primers RKOD-1 and RKOD-72 (S1 Table). The resulting PCR product was treated with T4 DNA polymerase at 12°C for 20 min in the presence of 1 mM dTTP and ligated into pHBM905A vector digested with *Not*I and *Cpo*I [12].

For expression in *E*. *coli*, the primers Kod-F and Kod-R and Kod-F and Kod-S-R were used to amplify KOD and KOD-Sso from the recombinant T-vectors. The resulting PCR products were ligated into pBAD-His-GFP vector digested with *Bfu* I [9].

### Expression and purification of RKOD in *Pichia pastoris*

For expression in *P*. *pastoris*, the plasmid pHBM-RKOD was linearized using *Sal*I and used to transform *P*. *pastoris* GS115 via electroporation (4 kΩ, 50μF, and 400 V). Transformants were screened on MD plates containing 0.4 mg/l biotin without histidine and identified by PCR using the primer pair RKOD-1 and RKOD-72. Recombinant *Pichia pastoris* GS115 bearing the target gene in its genome was incubated in 100 ml BMGY until the OD_600_ reached approximately 20. All cells were harvested by centrifugation and transferred to 25 ml of BMMY. A total of 1% (v/v) methanol was added every 24 h to induce target protein expression. Approximately 1 ml of cell culture was collected every 24 h and centrifuged at 6,000×*g* for 5 min to remove cells. After 168 h, an equal volume of each supernatant (15 μl) was loaded onto a 12% (w/v) polyacrylamide gel for SDS-PAGE according to the procedure of Laemmli [13], followed by staining with Coomassie Brilliant Blue G-250. The total protein concentration in each supernatant was measured using the Micro-BCA Protein Assay Reagent (Pierce, USA).

After induction with methanol for 120 h, the target protein was purified by centrifuging approximately 20 ml of BMMY cell culture at 6,000×g for 5 min. Then, the supernatant was immersed in a 75°C water orbital shaker and shaken for 30 min, cooled on ice for 20 min, and then centrifuged at 10,000×*g* at 4°C for 20 min. The clarified supernatant was collected and dialyzed in a Millipore 30-kDa cut-off membrane at 4°C to remove ions and salts. The sample was resuspended in 15 ml of Buffer A (20 mM Tris-HCl, 0.1 M NaCl, and 0.1 mM EDTA, pH 7.5) and centrifuged at 5000×*g* to achieve a final volume of 1 ml. This step was repeated twice.

### Expression and purification of KOD and KOD-Sso in *E*. *coli*

Plasmid encoding *KOD* and *KOD-Sso* was used to transform *E*. *coli* strain BL21 (Stratagene), which was grown in 500 ml of LB medium in the presence of carbenicillin to an OD600 of 0.5. Arabinose was added at a concentration of 1 mg/ml to induce expression from the *ara* promoter.

Cells were harvested 7 h later by centrifugation, and the pellet was resuspended in 20 ml of binding buffer (20 mM Tris-HCl, pH 7.9, 0.5 M NaCl, and 5 mM imidazole). The cells were disrupted by sonication, and the insoluble debris was removed by centrifugation. For purification, the cleared lysate was immersed in a 75°C water orbital shaker and shaken for 30 min, cooled on ice for 20 min, and then centrifuged at 10,000×*g* at 4°C for 20 min to remove the denatured *E*. *coli* proteins. A His-bind resin and His-bind buffer kit (Novagen, Madison, WI, USA) were used to purify the His-tagged protein according to Novagen’s instructions. The protein was eluted from the resin in 4 ml of elution buffer (20 mM Tris-HCl, pH 7.9, 100 mM imidazole, and 0.5 M NaCl). The protein concentration was measured using the Micro-BCA Protein Assay Reagent (Pierce, USA). The active fractions were pooled and dialyzed against storage buffer (20 mM Tris-HCl, 0.1 M NaCl, and 0.1 mM EDTA, pH 7.5) at 4°C overnight.

### DNA polymerase assays

DNA polymerase activity was assayed essentially as described by Grippo and Richardson [14]. The 50-μl reaction mixture contained 5 μl of 10×RKOD buffer (100 mM Tris-HCl, pH 8.8, 10 mM (NH_4_)_2_SO_4_, 100 mM KCl, 20 mM MgSO_4_, 0.1% Triton-100, and 1 mg/ml bovine serum albumin), 0.5 mM heat-denatured salmon sperm DNA, 0.20 mM each dCTP, dATP, dTTP, and dGTP, and 1 μCi of [α-32P]dCTP. The assay reactions were incubated at 72°C for 30 min and terminated on ice; 5-μl aliquots were then spotted onto ion-exchange paper discs (DE-81, Whatman). The spotted discs were dried, washed three times with 2x SSC buffer (300 mM sodium chloride and 30 mM trisodium citrate, pH 7.0) for 5 min each, washed once in 100% cold ethanol, and air-dried. The incorporated radioactivity was counted using a liquid scintillation counter. *KOD-Plus* DNA polymerase (0.5–2.5 units) was used as a positive control. One unit of RKOD DNA polymerase is defined as the amount of *RKOD* polymerase that incorporates 10 nmol dNTPs into a DE81-bound form at 72°C in 30 min.

The optimal reaction conditions for the recombinant *RKOD* DNA polymerase were used in the DNA polymerase assay as described previously. *RKOD* activity was measured under different pH, MgSO_4_ concentration, and temperature conditions.

### Staining Glycoproteins in SDS-Polyacrylamide Gels

Glycoprotein staining was performed using the Thermo Scientific Pierce Glycoprotein Staining Kit in accordance with the manufacturer’s recommendations. An equal amount of each sample was loaded onto two 12% (w/v) polyacrylamide gels. After SDS-PAGE, one gel was stained with Coomassie Brilliant Blue G-250, and the other was stained using the Glycoprotein Staining Kit.

### Thermostability analysis

Temperature stability was assayed as described by Dabrowski S, and J Kur [2]. Purified *RKOD*, KOD and KOD-Sso were heated at 95°C and 100°C from 1 h to 21 h. The same amount of enzyme was used to amplify an approximately 1-kb target fragment under the same reaction conditions. Each PCR reaction was performed in a 25-μl mixture containing 2.5 μl of 10× RKOD buffer (100 mM Tris-HCl, pH 8.8, 10 mM (NH_4_)_2_SO_4_, 100 mM KCl, 20 mM MgSO_4,_ 0.1% Triton-100, and 1 mg/ml bovine serum albumin), 10 pmol of primer P905-F and P1000-R (S1 Table), 0.01 μg of the pHBM905A plasmid, and 0.2 mM each dATP, dGTP, dCTP and dTTP. The PCR cycling parameters were as follows: 2 min of denaturation at 95°C, 25 cycles of 10 s at 95°C, 15 s at 56°C, and 30 s at 72°C, ending with an additional 7-min elongation at 72°C. Equal volumes of PCR products (4 μl) were analyzed by agarose gel electrophoresis.

### Fidelity of DNA polymerase

A PCR-based forward mutation assay was performed as described by Kelly S. Lundberg et al [15], except that “mannanase activity” was employed to measure the error rate of the *RKOD* DNA polymerase. The mannanase gene was amplified from pYEV–*man* [16] using the primers man-F and man-R (S1 Table) and cloned into the *BfuI* site of pBAD-His-GFP; the resulting construct is referred to as pBAD-mannanase. The plasmid pBAD-mannanase was amplified by inverse PCR [17] with the primer pair p-man-F and p-man-R (S1 Table) and contained a homologous sequence of 18 base pairs. PCR was performed with the *RKOD*, *KOD plus*, and *Prime Star* DNA polymerases under optimized conditions for each polymerase. The resulting PCR products were treated with *Dpn*I, subjected to agarose gel DNA purification, and transformed into *E*. *coli XL10-Gold*. These clones were transferred to LB plates containing 100 mM arabinose and 0.03% (m/v) trypan blue (which gives the plates a blue appearance) and 0.5% (m/v) Konjac Flour as the substrate for the mannanase. A clear zone was visible around mannanase-expressing colonies capable of hydrolyzing the Konjac glucomannan [16–18]. A single colony of *E*. *coli XL10-Gold* that contained the plasmid pBAD-man was used as a positive control, and a single colony of *E*. *coli XL10-Gold* that contained the plasmid pBAD-His-GFP was used as a negative control. Hydrolysis holes with different sizes were observed and measured after 12 hours of culture. Error rates were calculated based on the sizes of the hydrolysis holes. Clones that produced much larger or smaller hydrolysis holes were considered mutants.

### PCR efficiency assay

In this experiment, plasmid pHBM905A targets (2–9 kb) were amplified using a range of extension times to compare the efficiencies of *RKOD*, *KOD* and *KOD-S*. The plasmid pHBM905A was used as the template. The primer pairs P905-F and P2000-R (for 2 kb), P905-F and P4000-R (for 4 kb), P905-F and P6000-R (for 6 kb) and P905-F and P9000-R (for 9 kb) (S1 Table) were designed to amplify targets with lengths of 2 kb, 4 kb, 6 kb, and 9 kb, respectively. Each PCR reaction was performed in a 50-μl mixture containing 5 μl of 10× RKOD buffer (100 mM Tris-HCl, pH 8.8, 10 mM (NH_4_)_2_SO_4_, 100 mM KCl, 20 mM MgSO_4_, 0.1% Triton-100, and 1 mg/ml bovine serum albumin), 10 pmol each primer, 0.01 μg of the pHBM905A plasmid, 0.2 mM each dATP, dGTP, dCTP and dTTP, and purified RKOD, KOD and KOD-Sso. The PCR cycling parameters were as follows: 2 min denaturation at 95°C, 25 cycles of 10 s at 95°C, 15 s at 56°C, and 30–50 sec at 72°C, ending with an additional 7-min elongation at 72°C. Equal volumes of PCR products (4 μl) were analyzed using 1% agarose gel electrophoresis.

## Results

### Optimization and synthesis of the *RKOD* gene

The *KOD* gene of *Pyrococcus sp*. *strain KOD1* contains a 5013-bp open reading frame (GenBank: D29671); the entire mature *KOD* DNA polymerase encodes a 90-kDa protein. The *Sso7d* gene from *S*. *solfataricus P2* (Gene ID: 1454006) is 207 bp in length and encodes a 7-kD protein. These sequences were modified based on the codon usage bias of *P*. *pastoris* without changing the amino acid sequence and synthesized by overlapping PCR. The *Sso7D* gene was fused to the C-terminus of *KOD*. For expression in *P*. *pastoris*, the *KOD-Sso* fragment was cloned into the pHBM905A vector fused with the MF-α leader sequence. The recombinant plasmid was confirmed by DNA sequencing and designated pHBM*-RKOD*. For expression in *E*. *coli*, *KOD* and *KOD-Sso* were cloned into the pBAD-His-GFP vector fused with the 6xHis tag under the control of the *ara* promoter. The recombinant plasmids were confirmed by DNA sequencing and termed pBAD-*KOD* and pBAD-*KOD-S*, respectively.

### Expression and purification of the recombinant KOD DNA polymerase

**Fig 1:**
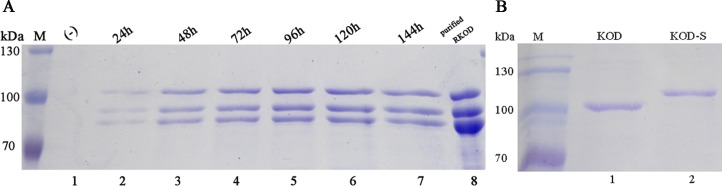
SDS-PAGE analysis of secreted RKOD in the cell culture supernatants of the shake flasks and soluble KOD and KOD-S expressed in *E*. *coli*. (A). M, protein molecular weight marker (the size of each band is indicated on the left); lane 1, cell culture supernatant (15 μl) of the strain bearing the pHBM905A plasmid (negative control); lanes 2–7, cell culture supernatants (15 μl) collected from days 1–6, respectively; and lane 8, purified RKOD; (B). M, protein molecular weight marker (the size of each band is indicated on the left); lane 1, purified KOD; lane 2, purified KOD-S.

Positive colonies were selected randomly to assess the expression of RKOD. SDS-PAGE of the supernatants revealed the presence of three bands from the first day of induction. (Fig 1). All bands were identified as the target protein RKOD by mass spectrometry (MS) (data not shown). The total amount of protein in the supernatant was 150, 181, 192 and 230 mg/l on days 1, 2, 3, 4, respectively. The expression of RKOD reached a maximum level at 120 h, with a protein concentration of approximately 250 mg/l. RKOD expression decreased thereafter to 239 mg/l at 144 h. These results demonstrated the successful expression of RKOD in *P*. *pastoris*. The recombinant protein was designated RKOD. The fermentation supernatant was purified, and the protein concentration was 180 mg/l after purification.

Soluble KOD and KOD-S expressed in *E*. *coli* were purified by Ni-chelate affinity chromatography. The protein concentrations of KOD and KOD-S were 25 mg/l and 20 mg/l after purification, respectively.

### Activity assessment of recombinant RKOD

The enzymatic activities of purified RKOD, KOD and KOD-S were approximately 19,384 U/mg, 20,500 U/mg and 20,100 U/mg, respectively. These results indicate that Sso7d fusion did not negatively affect the catalytic activity of the KOD DNA polymerase.

**Fig 2:**
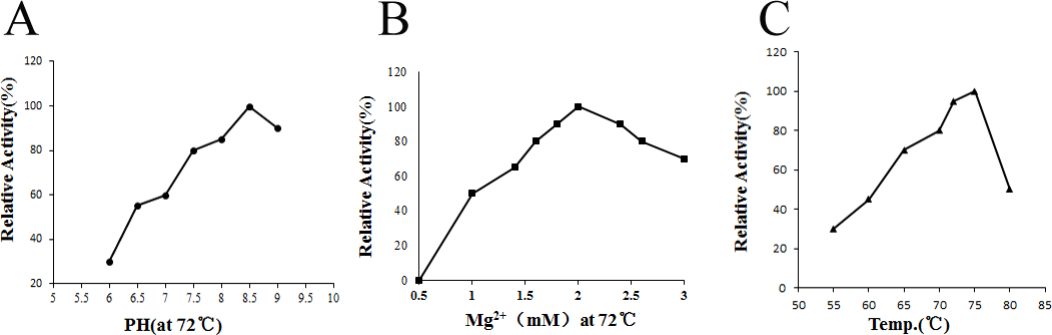
Effects of temperature, pH and Mg^2+^ on RKOD DNA polymerase activity. (A) Effect of pH on DNA polymerase activity. (B) Effect of Mg^**2+**^ on DNA polymerase activity. (C) Effect of temperature on DNA polymerase activity. The standard assay described in the text was performed with 1 U of purified DNA polymerase. These experiments were repeated for 3 times.

Next, we determined the characteristics of the RKOD DNA polymerase (i.e., optimum pH, temperature, and required concentration of metal ions). RKOD exhibited maximum activity at pH 8.5 (Fig 2A). Similar to all other DNA polymerases, RKOD required a divalent cation as a cofactor; optimal activity was obtained with 2.0 mM MgSO_4_ (Fig 2B). The optimal temperature for the catalytic activity of *RKOD* was (Fig 2C) 70–75°C.

### Glycosylation analysis of RKOD

**Fig 3:**
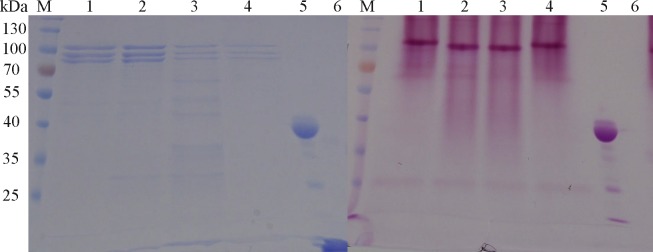
Staining of Glycoproteins in SDS-Polyacrylamide Gels. (A). SDS-PAGE of RKOD with or without digestion with Endo H. M, protein molecular weight marker (the size of each band is indicated on the left); lane 1, RKOD; lane 2, RKOD treated with Endo H; lane 3, denatured RKOD treated with Endo H; lane 4, denatured RKOD; lane 5, positive control (Horseradish Peroxidase) from the kit; and lane 6, negative control (Soybean Trypsin Inhibitor) from the kit. (B). Glycoprotein staining of RKOD with or without Endo H digestion. All samples were loaded in the same order as A.

According to the online Prediction Servers NetNGlyc 1.0 and NetOGlyc 4.0 (http://www.cbs.dtu.dk/services/NetNGlyc/ and http://www.cbs.dtu.dk/services/NetOGlyc/), *RKOD* has one putative N-linked glycosylation site (NSV) and six putative O-linked glycosylation sites. Thus, the variation in the apparent molecular mass of *RKOD* observed by SDS-PAGE may be attributable to glycosylation. Endo H is commonly used to identify the composition of the glycan portion of N-glycosylated proteins [19–20]. To test our hypothesis, RKOD was treated with Endo H under both native and denaturing condition, followed by staining with the GelCode Glycoprotein Staining Kit to detect glycoprotein sugar moieties. As clearly demonstrated in Fig 3, the upper band of RKOD appeared as a magenta band, indicating glycosylation of RKOD.

### Temperature stability assay

**Fig 4:**
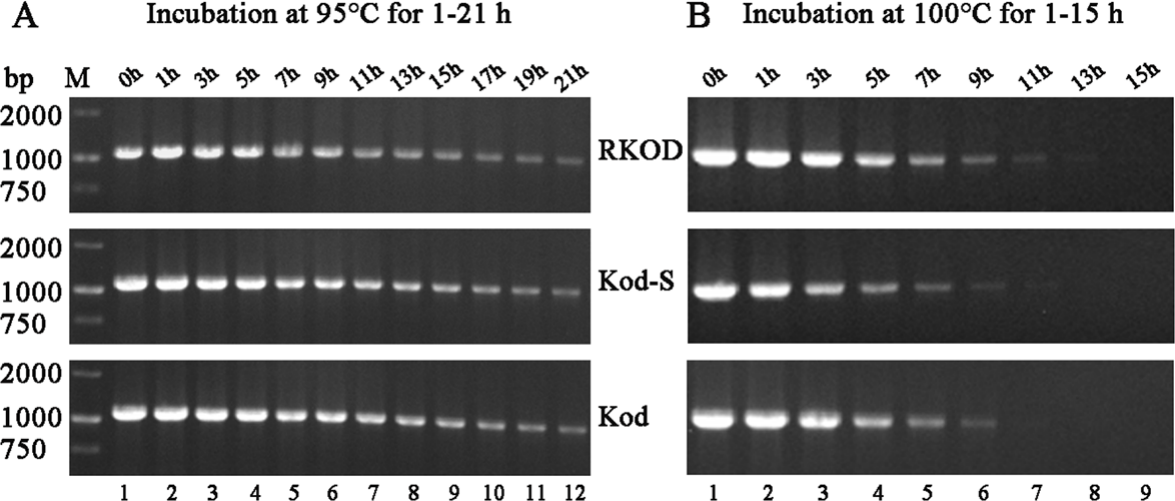
Thermostability of RKOD expressed in *Pichia pastoris*. Residual activity was assayed by PCR after incubation at 95°C or 100°C. (A). Thermostability of the *KOD*, *KOD-S* and *RKOD* DNA polymerase at 95°C. M, DL2000 marker (the size of each band is indicated on the left); lane 1, PCR with untreated *KOD*, *KOD-S* and *RKOD* (positive control); lanes 2–12, PCR with *KOD*, *KOD-S* and *RKOD* incubated at 95°C for 1–21 h. (B). Thermostability of the *KOD*, *KOD-S* and *RKOD* DNA polymerase at 100°C. Lane 1, PCR with untreated *KOD*, *KOD-S* and *RKOD* (positive control); lanes 2–9, PCR with *KOD*, *KOD-S* and *RKOD* incubated at 100°C for 1–15 h.

Protein glycosylation is one of the most common covalent post-translational modifications and has a significant effect on the structure and function of proteins, particularly thermostability [21–22]. A PCR-based assay was used to analyze the thermostability of *KOD*, *KOD-S* and *RKOD*. Gel imaging analysis revealed that *KOD*, *KOD-S* and *RKOD* retained their polymerase activity after incubation at 95°C for 21 h (Fig 4A). Following incubation at 100°C for 11 h, (Fig 4B), *RKOD* retained polymerase activity, but the activities of *KOD* and *KOD-S* were reduced. These results indicate *RKOD* is more thermostable than *KOD* and *KOD-S*, possibly due to the glycosylation of RKOD. However, the thermal stability profile of the *KOD-S* fusion protein was nearly identical to that of *KOD*. These results demonstrate that the fusion of Sso7d to the polymerase does not negatively affect the thermal stability of the polymerase domain.

### PCR error rate of the RKOD DNA polymerase

High-fidelity PCR enzymes are valuable for minimizing the introduction of amplification errors in products that will be cloned, sequenced, and expressed [23]. Therefore, maintaining the high fidelity of the *RKOD* polymerase obtained from different expression systems is important. *RKOD* expressed in *P*. *pastoris* had an error rate of 0.38%, similar to the error rates of commercial high-fidelity PCR enzymes (Table 1).

**Table 1:** Comparison of the fidelities of thermostable DNA polymerases.

Enzymes	No. of clear zones		Mutation frequency^*b*^ (%)
	Mutant	Total	-
RKOD	20	5250	0.38
KOD plus	16	5000	0.32
Prime Star	15	5300	0.28

^*a*^Mutant: the clear zone of the colonies was much larger/smaller than that of the positive control

Total: all colonies on the plate.

^*b*^Mutant colonies/total colonies.

### PCR efficiency assay

**Fig 5:**
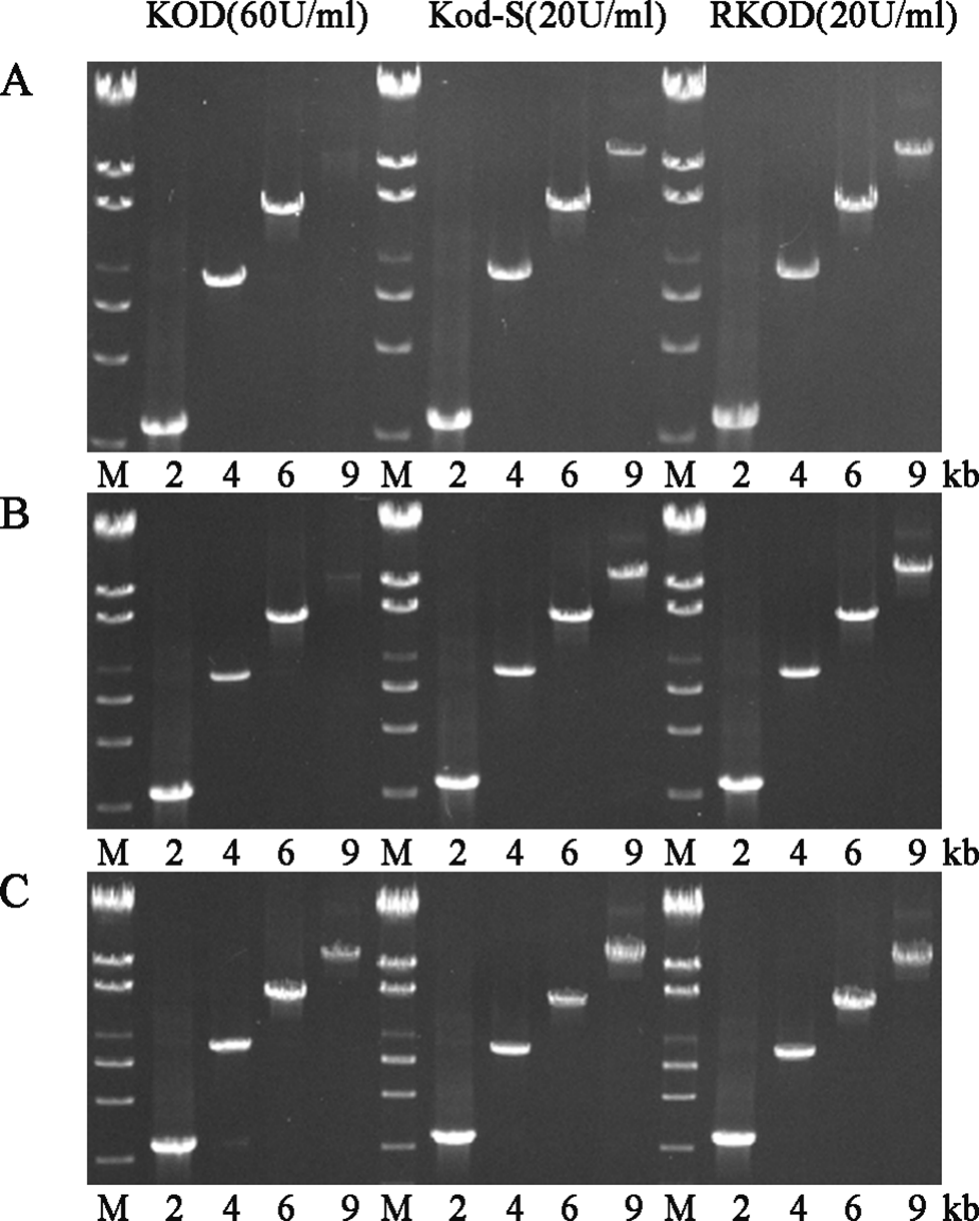
Comparison of the PCR efficiencies of *KOD*, *KOD-S* and *RKOD*. The plasmid pHBM905A was used as the template, and the sizes of the amplicons are indicated at the bottom. M, λ/EcoR T14 marker; lanes 1–4, the target lengths of 2 kb, 4 kb, 6 kb, and 9 kb are marked. PCR buffer 10 mM Tris-HCl, pH 8.8, 1 mM (NH_4_)_2_SO_4_, 10 mM KCl, 2 mM MgSO_4_, 0.01% Triton-100, and 0.1 mg/ml bovine serum albumin, 10 pmol each primer, 0.01 μg of the pHBM905A plasmid, 0.2 mM each dNTPs. The cycling protocol was 95°C for 2 min; 25 cycles of 95°C for 10 s, 56°C for 15 s and 72°C for 30 s (A) or for 40 s (B) or for 50 s (C).

The major uses of thermostable DNA polymerases are for in vitro amplification of DNA fragments and DNA sequencing. The ultimate goal of improving the processivity of thermostable DNA polymerases is to improve their performance in vitro, such as in PCR or cycle sequencing applications [7]. Because an increase in processivity allows the polymerase to incorporate more nucleotides per binding event, it should also enable more efficient in vitro replication of the template strand during each PCR cycle. Consequently, shorter extension times may be required to amplify the same target or a much longer target could be amplified with the same extension time. In this experiment, the plasmid pHBM905A (2–9 kb) was amplified using a range of extension times to compare the efficiencies of *KOD*, *KOD-S* and *RKOD* (Fig 5). A 50-sec/cycle extension time was required to amplify a 9-kb target when 60 U/ml *KOD* was used. By contrast, when 20U/ml *KOD-S* and *RKOD* were used the same 9-kb target could be amplified with a 30 s/cycle extension time. These findings demonstrate that when Sso7d is fused to KOD, a significant enhancement of processivity is achieved regardless of the starting processivity of the enzyme. When *KOD-S* and *RKOD* are tested in PCR amplifications, a clear advantage over *KOD* is observed. Not only is much less enzyme required, but a much shorter extension time can be used.

## Discussion

PCR is a standard industry process that is commonly used to produce linear DNA due to its significant facility and higher efficiency compared to bacterial fermentation. The applications of PCR products include DNA vaccines, RNA delivery, and gene therapy [24–28]. However, DNA polymerase is a costly component of PCR systems and can restrict the use of PCR.

Vandalia Research Company is capable of providing milligram or gram quantities of PCR-amplified DNA products using the Triathlon DNA method [29]. However, the expense of the DNA polymerase prevents the production of large quantities of DNA instantly for clinical consumption, particularly for industrial applications. Therefore, we sought an easy, inexpensive and rapid method to obtain a high-efficiency DNA polymerase.

Most DNA polymerases are expressed in *E*. *coli*. However, the low expression level and cumbersome purification protocol [1–3] prevents the rapid production of large quantities of DNA polymerase. As an inexpensive and effective strategy to obtain DNA polymerase, we expressed DNA polymerase in the yeast *P*. *pastoris*, which is frequently used to express and secret proteins. This yeast expression system has several advantages, including the availability of the strong methanol-induced alcohol oxidase (AOX1) promoter, the high expression level, and the convenient purification procedure. Moreover, *P*. *pastoris* can accommodate high cell densities and post-translational modifications of foreign proteins [30–32].

In this study, we constructed a recombinant *P*. *pastoris* strain expressing the DNA polymerase designated *RKOD*. We obtained approximately 250 mg of this recombinant DNA polymerase per liter, with an activity of approximately 19,384 U/mg. This study is the first to report the high expression of a DNA polymerase in *P*. *pastoris*. Evaluation of the characteristics of the RKOD DNA polymerase revealed that RKOD had excellent elongation rates, fidelity and thermostability, consistent with a previous study [33]. These results suggest that the RKOD DNA polymerase is suitable for long, accurate, and time-saving PCR [34–36]. We plan to test the ability of the RKOD DNA polymerase in a large-scale PCR of 200 milliliters or more. Preliminary results are promising (data not shown). Our results will contribute to the expansion of the applications of PCR. The successful expression of the RKOD DNA polymerase in a yeast system will reduce the cost of DNA polymerase production, shorten the procedure, and simplify the purification. This process serves as an excellent foundation for obtaining a large amount of high-fidelity DNA polymerase with good thermostability. The results of this study will also expand the applications of RKOD. For example, we can establish a large-scale PCR for producing linear DNA in response to new outbreaks of infectious disease. The successful expression of the recombinant DNA polymerase *RKOD* in the *P*. *pastoris* system will increase the advantages of the use of PCR in research.

## Supporting Information

S1 Table Primers used for PCR in this study (DOCX)
