# Supplementary material for: Expression and Characterization of the RKOD DNA Polymerase in Pichia pastoris
Source: PLoS One. 2015 Jul 2;10(7):e0131757. doi: 10.1371/journal.pone.0131757 (PMC4489709; doi:10.1371/journal.pone.0131757)
Supplement: S1 Table — (DOCX) [file pone.0131757.s001.docx]

**S1 Table. Primers used for PCR in this study**

| Primers | oligonucleotide Sequences (5'→3') |
| --- | --- |
| RKOD-1 | gatcatgatcctcgacactgactacataaccgaggatgg |
| RKOD-2 | gttttccttcttgaaaattcttatgacaggctttccatcctcggttatgtagtca |
| RKOD-3 | gtcataagaattttcaagaaggaaaacggcgagtttaagattgagtacgaccgga |
| RKOD-4 | ccttcaggagggcgtagaagtagggttcaaaagtccggtcgtactcaatcttaaa |
| RKOD-5 | ttctacgccctcctgaaggacgattctgccattgaggaagtcaagaagataaccg |
| RKOD-6 | cttaaccgttacaaccgtcccgtgcctctcggcggttatcttcttgacttcctca |
| RKOD-7 | ggacggttgtaacggttaagcgggttgaaaaggttcagaagaagttcctcgggag |
| RKOD-8 | gatgagtaaagtagagtttccagacctcaactggtctcccgaggaacttcttctg |
| RKOD-9 | gtctggaaactctactttactcatccgcaggacgtcccagcgataagggacaaga |
| RKOD-10 | actcgtagatgtcaataactgctggatgctctcgtatcttgtcccttatcgctgg |
| RKOD-11 | agcagttattgacatctacgagtacgacatacccttcgccaagcgctacctcata |
| RKOD-12 | gctcctcgtcgccttccattggcactaatcccttgtctatgaggtagcgcttggc |
| RKOD-13 | gaaggcgacgaggagctgaaaatgctcgccttcgacattgaaactctctaccatg |
| RKOD-14 | aaggattggcccctcggcgaactcctcgccctcatggtagagagtttcaatgtcg |
| RKOD-15 | ccgaggggccaatccttatgataagctacgccgacgaggaaggggccagggtgat |
| RKOD-16 | cgacgtcaacgtaggggagatccacgttcttccaagttatcaccctggccccttc |
| RKOD-17 | tcccctacgttgacgtcgtctcgacggagagggagatgataaagcgcttcctccg |
| RKOD-18 | ttatgagaacgtccgggtctttctccttcacaacacggaggaagcgctttatcat |
| RKOD-19 | agacccggacgttctcataacctacaacggcgacaacttcgacttcgcctatctg |
| RKOD-20 | cgaagtttattccgagcttttcacagcgctttttcagataggcgaagtcgaagtt |
| RKOD-21 | tgaaaagctcggaataaacttcgccctcggaagggatggaagcgagccgaagatt |
| RKOD-22 | ccttcacttcgacggcaaacctgtcgcccatcctctgaatcttcggctcgcttcc |
| RKOD-23 | tttgccgtcgaagtgaagggacggatacacttcgatctctatcctgtgataagac |
| RKOD-24 | caagcgtgtatgtgggcaggtttatcgtccgtcttatcacaggatagagatcgaa |
| RKOD-25 | tgcccacatacacgcttgaggccgtttatgaagccgtcttcggtcagccgaagga |
| RKOD-26 | tttcccaggctgtggttatttcctcagcgtaaaccttctccttcggctgaccgaa |
| RKOD-27 | aaataaccacagcctgggaaaccggcgagaaccttgagagagtcgcccgctactc |
| RKOD-28 | ccttcccaagctcgtatgtgaccttcgcatcttccatcgagtagcgggcgactct |
| RKOD-29 | cacatacgagcttgggaaggagttccttccgatggaggcccagctttctcgctta |
| RKOD-30 | tgctggagcgggagacgtcccagagggactggccgattaagcgagaaagctgggc |
| RKOD-31 | gtctcccgctccagcactggcaacctcgttgagtggttcctcctcaggaaggcct |
| RKOD-32 | tcatcgggcttgttcggggccagctcattcctctcataggccttcctgaggagga |
| RKOD-33 | cccgaacaagcccgatgaaaaggagctggccagaagacggcagagctatgaagga |
| RKOD-34 | ctcccacaaccctctctcgggctcttttacatagcctccttcatagctctgccgt |
| RKOD-35 | cgagagagggttgtgggagaacatagtgtacctagattttagatccctgtacccc |
| RKOD-36 | tccggcgagacgttgtgggtgatgatgattgaggggtacagggatctaaaatcta |
| RKOD-37 | cacaacgtctcgccggatacgctcaacagagaaggatgcaaggaatatgacgttg |
| RKOD-38 | agtccttgcagaagcggtggccgacctgtggggcaacgtcatattccttgcatcc |
| RKOD-39 | accgcttctgcaaggacttcccaggatttatcccgagcctgcttggagacctcct |
| RKOD-40 | ggccttcatcttcttctttatcttctgcctctcctctaggaggtctccaagcagg |
| RKOD-41 | gaagataaagaagaagatgaaggccacgattgacccgatcgagaggaagctcctc |
| RKOD-42 | tttgccaggatcttgatggccctctgcctgtaatcgaggagcttcctctcgatcg |
| RKOD-43 | gccatcaagatcctggcaaacagctactacggttactacggctatgcaagggcgc |
| RKOD-44 | aggccgttacgctctctgcacactccttgcagtaccagcgcgcccttgcatagcc |
| RKOD-45 | cagagagcgtaacggcctggggaagggagtacataacgatgaccatcaaggagat |
| RKOD-46 | gctgtagattaccttaaagccgtacttttcctctatctccttgatggtcatcgtt |
| RKOD-47 | cggctttaaggtaatctacagcgacaccgacggattttttgccacaatacctgga |
| RKOD-48 | catagccttctttttgacggtttcagcatcggctccaggtattgtggcaaaaaat |
| RKOD-49 | aaccgtcaaaaagaaggctatggagttcctcaagtatatcaacgccaaacttccg |
| RKOD-50 | gtagaagccctcgtactcgagctcaagcgcgcccggaagtttggcgttgatatac |
| RKOD-51 | tcgagtacgagggcttctacaaacgcggcttcttcgtcacgaagaagaagtatgc |
| RKOD-52 | gcgcgttgttatcttgccttcctcgtctatcaccgcatacttcttcttcgtgacg |
| RKOD-53 | ggcaagataacaacgcgcggacttgagattgtgaggcgtgactggagcgagatag |
| RKOD-54 | gcaaagcttcaagaaccctcgcctgcgtctctttcgctatctcgctccagtcacg |
| RKOD-55 | gagggttcttgaagctttgctaaaggacggtgacgtcgagaaggccgtgaggata |
| RKOD-56 | ctcgtacttgctcagcttttcggtaacttctttgactatcctcacggccttctcg |
| RKOD-57 | gaaaagctgagcaagtacgaggttccgccggagaagctggtgatccacgagcaga |
| RKOD-58 | ggaccggttgccttgtagtcctttaaatccctcgttatctgctcgtggatcacca |
| RKOD-59 | ctacaaggcaaccggtccccacgttgccgttgccaagaggttggccgcgagagga |
| RKOD-60 | cacgatgtagcttatcaccgttccagggcgtattttgactcctctcgcggccaac |
| RKOD-61 | acggtgataagctacatcgtgctcaagggctctgggaggataggcgacagggcga |
| RKOD-62 | gtacttgtgcttcgtcgggtcgaactcgtcgaacggtatcgccctgtcgcctatc |
| RKOD-63 | cccgacgaagcacaagtacgacgccgagtactacattgagaaccaggttctccca |
| RKOD-64 | gcggtaaccgaaggctctcagaattctctcaacggctgggagaacctggttctca |
| RKOD-65 | gagccttcggttaccgcaaggaagacctgcgctaccagaagacgagacaggttgg |
| RKOD-66 | ccatagttcccttcggcttcagccaagcactcaaaccaacctgtctcgtcttctg |
| RKOD-67 | gaagccgaagggaactatggcgaccgtgaaatttaaatataaaggcgaagaaaaa |
| RKOD-68 | acttttttaattttgctaatatccacttctttttcttcgcctttatatttaaatttca |
| RKOD-69 | gaagtggatattagcaaaattaaaaaagtgtggcgcgtgggcaaaatgattagct |
| RKOD-70 | gcggccggttttgccgccgccttcatcataggtaaagctaatcattttgcccacg |
| RKOD-71 | ggcaaaaccggccgcggcgcggtgagcgaaaaagatgcgccgaaagaactgctgc |
| RKOD-72 | ggccattattttttctgtttttccagcatctgcagcagttctttcggcg |
| Kod-F | atgatcctcgacactgactacataacc |
| Kod-R | cacaattagttcccttcggcttcagcc |
| Kod-S-R | cacaattttattttttctgtttttccagcatctg |
| P905-F | agatctaacatccaaagacgaaaggttg |
| P1000-R | agtgttgactggagcagctaatgcg |
| P2000-R | gtagcgttgccaatgatgttacagatg |
| P4000-R | agaaggatggtacccttaccccaga |
| P6000-R | cgaactggatctcaacagcggtaa |
| P9000-R | agatcttgagataaatttcacgtttaaa |
| Man-F | atgggggagttgcatttgtttaaga |
| Man-R | ttactcaacgattggcgttaaagaatc |
| p-man-F | tctgcgcgtaatctgctgcttgc |
| p-man-R | cagcagattacgcgcagaaaaaaagg |

Note: The underlined RKOD-1 and RKOD-72 sequences matched the sticky ends generated by *Cpo*I and *Not*I, respectively.

The underlined p-man-F and p-man-R are the [homologous](app:ds:homologous) [sequence](app:ds:sequence).
